# *Innovation in Aging*’s Calling Card: *Translational Significance*

**DOI:** 10.1093/geroni/igaa064

**Published:** 2021-01-01

**Authors:** Steven M Albert

**Affiliations:** Department of Behavioral and Community Health Sciences, Graduate School of Public Health, University of Pittsburgh, Pennsylvania, USA

I am honored to take the reins of *Innovation in Aging (IA)* as its second Editor-in-Chief. In its first 4 years, *IA* has already made its mark in aging science. We have published more than 135 papers and put together highly cited special issues and sections in caregiving dynamics, Latinx health, public health and aging, and mental health among older adults in minority groups. *IA* is indexed in PubMed, the Emerging Sources Citation Index (Web of Science), the Directory of Open Access Journals, and SCOPUS. We continue to see submissions at an expanding pace and, on average, get first decisions back to authors within 25 days. With increasing citations, we are on track to receive an impressive journal impact factor.

However, in science, we do not rest on our laurels. What’s next for *IA*?

Translation in research on aging is the hallmark of *IA*. Each article in *IA* requires a statement on translational significance prominently placed in a blue box near the title. This is not window dressing but rather forces authors to consider the implications of their research for *changing* some component of aging, enhancing our ability to address a challenge posed by aging bodies, minds, relationships, or societies. This is a tall order. It will likely require more “convergence science,” research that crosses levels and integrates “biological, psychological, sociocultural, and environmental data” (Eyre et al., 2015). This research necessarily draws upon multiple disciplines and a wide range of methodologies (Albert, 2020). Fundamentally, *IA* seeks research that draws on multiple disciplines to solve problems that stand in the way of optimal aging.

*IA* will continue to build on its track record in convergence aging science. Some of our most prominent examples include a systems science approach to human capital to rethink engagement over the life span (Morrow-Howell et al., 2017), research on within-family differences to link early parent–child relationships and family dynamics in later life (Pillemer & Gilligan, 2018), and assessment of the built environment and human factors to distinguish aging effects and disability (Mitzner et al., 2018). *IA* offers a venue for people to think outside of the box as they complete our blue box for translational significance. Readers can expect special issues explicitly addressing interdisciplinary research, in which multiple authors from different disciplines will collaborate on topics in gerontology.

*IA* will also exploit the flexibility of open-access publishing. Open access will become increasingly more prominent in all disciplines, as it should, because this approach to publishing is consistent with the open data and open science movement. Open access is now required in the European Union and by an increasing number of funders. The challenge is expense to authors and confusion spread by predatory journals that have seized on open-access publishing. To confront this challenge, we need to educate authors about the benefits of open access, including increased dissemination and readership, but foundations and academic departments also need to set aside funds to cover publishing charges. Oxford University Press appropriately provides subsidies for scholars based in lower- and middle-income economies. The open-access format also offers other benefits, such as the absence of limits on pages or issues. I hope we can also use the open access model to develop interactive graphics and ways to “personalize” articles through slide downloads, chats with authors, and social media efforts.

*IA* will continue to rely on key partners to help bring out the best science from our authors. These include our associate editors and editorial and advisory boards for timely expert review and suggestions to carry out *IA*’s mission of interdisciplinary translational research, but also researchers who respond to the call to peer review, the GSA editorial management team, and Oxford University Press. As an editor, I have a new appreciation for the sophisticated machinery required to publish timely, high-quality research.

Lastly, an editor’s job is to encourage science and also the scientists. In particular, *IA* will reach out to junior scholars and help them develop their work to make it ready for publication. This is an old responsibility of editors that unfortunately may have gotten lost in the high-paced digital climate we now inhabit. Many of us have benefited from such an editor, and it is a tradition we should encourage. Along with *IA* associate editors, I would like to encourage support for early and new investigators through clear suggestions in response letters on ways to improve the science, helpful guidance on revisions, pairing with senior scholars for guidance, or just handholding over multiple revisions. This level of involvement is part of our job.

## References

[CIT0001] Albert, S. M (2020). Convergence gerontology: Rethinking translation in research on aging. Innovation in Aging, 4(2), igaa003. doi:10.1093/geroni/igaa003 32175482 PMC7060661

[CIT0002] Eyre, H. A., Lavretsky, H., & Insel, T. R. (2015, November 13). Convergence science: Shaping 21st century psychiatry. Psychiatric Times https://www.psychiatrictimes.com/schizophrenia/convergence-science-shaping-21st-century-psychiatry

[CIT0003] Mitzner, T. L., Sanford, J. A., & Rogers, W. A (2018). Closing the capacity–ability gap: Using technology to support aging with disability. Innovation in Aging, 2(1), igy008. doi:10.1093/geroni/igy008 30480132 PMC6176980

[CIT0004] Morrow-Howell, N., Halvorsen, C. J., Hovmand, P., Lee, C., & Ballard, E (2017). Conceptualizing productive engagement in a system dynamics framework. Innovation in Aging, 1(1), igx018. doi:10.1093/geroni/igx018 30480112 PMC6177040

[CIT0005] Pillemer, K., & Gilligan, M (2018). Translating basic research on the aging family to caregiving intervention: The case of within-family differences. Innovation in Aging, 2(1), igx035. doi:10.1093/geroni/igx035 30480127 PMC6177031

